# Clear cell adenocarcinoma of the cervix in a perimenopausal woman: a case report and immunohistochemical study

**DOI:** 10.1007/s12672-026-04699-6

**Published:** 2026-02-27

**Authors:** Rong Liu, Hanxia Jiang, Shuxian Feng, Hui Tian

**Affiliations:** Department of Gynecology, The 940th Hospital of Joint Service Support Force of Chinese People’s Liberation Army, Lanzhou, 730030 Gansu China

**Keywords:** Uterine cervix, Clear cell adenocarcinoma, Immunohistochemistry, Perimenopausal

## Abstract

We report a rare case of sporadic cervical clear cell adenocarcinoma (CCAC) in a 46-year-old perimenopausal woman without the classic risk factors of in utero diethylstilbestrol (DES) exposure or high-risk human papillomavirus (HPV) infection, who presented with abnormal vaginal bleeding. Imaging revealed a bulky, barrel-shaped cervix with a large mass (measuring 4.8 × 6.7 × 3.1 cm), classified as FIGO stage IB3 based on pelvic examination and imaging findings. The patient underwent radical surgery, and the diagnosis was confirmed histologically and supported by a definitive immunohistochemical profile: the tumor was positive for PAX-8 and napsin-A, showed only partial positivity for p16, and exhibited a high Ki-67 index (70%), consistent with an HPV-independent clear cell carcinoma. This case underscores the sporadic, HPV-independent form of CCAC and highlights the critical role of immunohistochemistry in establishing the diagnosis, particularly in clinically atypical presentations.

## Introduction

Cervical cancer is predominantly histologically categorized into squamous cell carcinoma (70–80% of cases) and adenocarcinoma (20–25% of cases) [1]. Among the rare subtypes of adenocarcinoma, cervical clear cell adenocarcinoma (CCAC) represents a distinct entity, accounting for only 1% to 4% of all cervical malignancies [1, 2]. Although historically associated with in utero diethylstilbestrol (DES) exposure, its immunophenotype remains incompletely characterized [3, 4]. Patients with prenatal DES exposure typically present between 15 and 31 years with tumors often involving the external cervix and upper anterior vaginal wall, while sporadic cases (without DES exposure) predominantly occur in women aged 65–80 years with endocervical involvement. Herein, we report a rare case of CCAC in a 46-year-old perimenopausal woman and present a detailed analysis of its immunohistochemical features.

## Case report

A 46-year-old perimenopausal woman presented to a local hospital with a chief complaint of abnormal vaginal bleeding for over ten days. She had no intrauterine exposure to diethylstilbestrol, no significant comorbidities, and no family history of hereditary cancer syndromes. Gynecological examination revealed an exophytic cervical mass. A pathological biopsy confirmed a malignant cervical tumor, and testing for human papillomavirus (HPV) was negative.

**Fig. 1:**
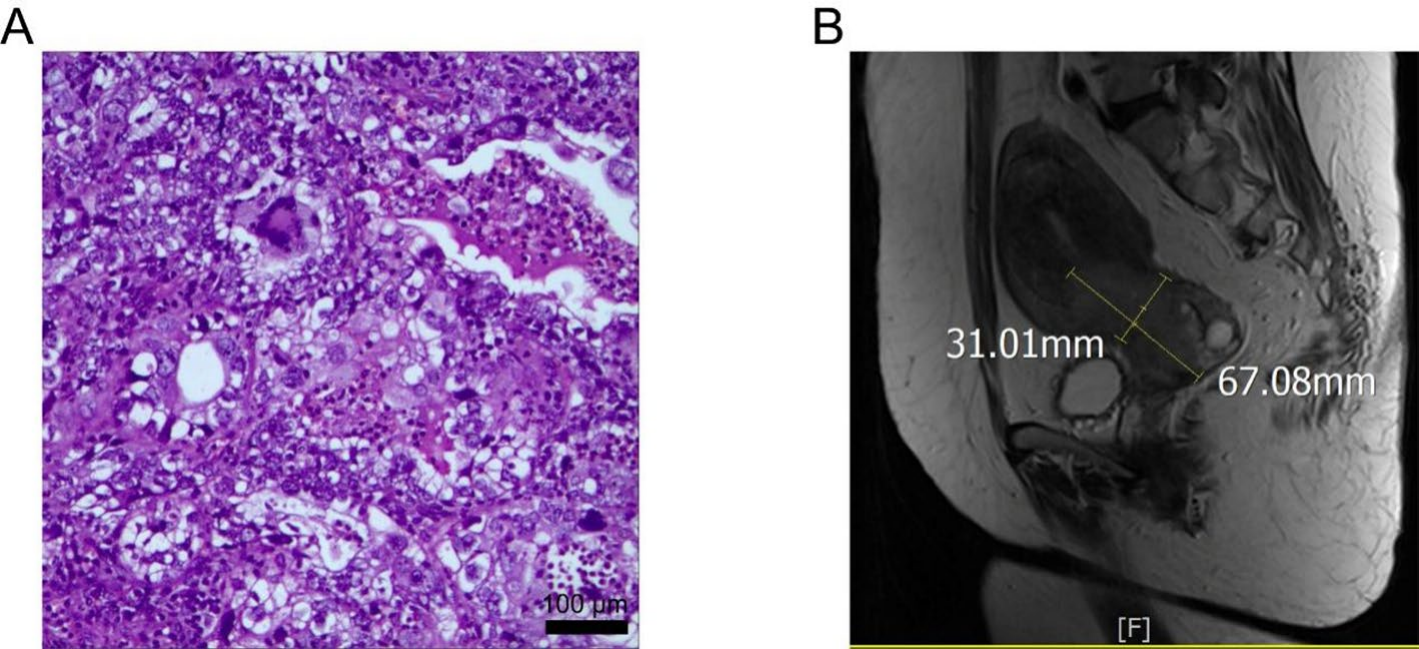
Histopathology and magnetic resonance imaging (MRI) findings. **A **Representative histology of cervical clear cell adenocarcinoma, low-power view illustrating a mixture of papillary and tubulocystic patterns. Scale bar = 100 µm. **B** MRI reveals an enlarged uterus (15.0 × 8.5 × 6.0 cm) with a mass (arrow) arising from the anterior cervical lip. The lesion measures 4.8×6.7×3.1 cm at its largest cross-section

In August 2025, she was referred to our hospital for further management. Gynecological examination confirmed a barrel-shaped, indurated cervix with necrotic tissue obscuring the external os. No obvious parametrial or uterosacral ligament involvement was detected. The uterus was enlarged, comparable to a 14–16 weeks gestation. Histopathological review of the biopsy slides at our institution revealed invasive carcinoma composed of irregular glandular, cystic, and papillary structures, consistent with a diagnosis of cervical clear cell carcinoma (Fig. 1a). Abdominal magnetic resonance imaging (MRI) demonstrated a mass measuring 4.8 × 6.7 × 3.1 cm arising from the anterior lip of the cervix. The tumor was confined to the cervix without invasion of the vagina or parametrial tissues. According to the International Federation of Gynecology and Obstetrics (FIGO) 2018 staging system for cervical cancer, a tumor with a maximum diameter > 4 cm that remains confined to the cervix is classified as stage IB3. Serum tumor markers were elevated, with CA-125 at 249.7 U/mL and CA19-9 at 227.3 U/mL.

**Fig. 2:**
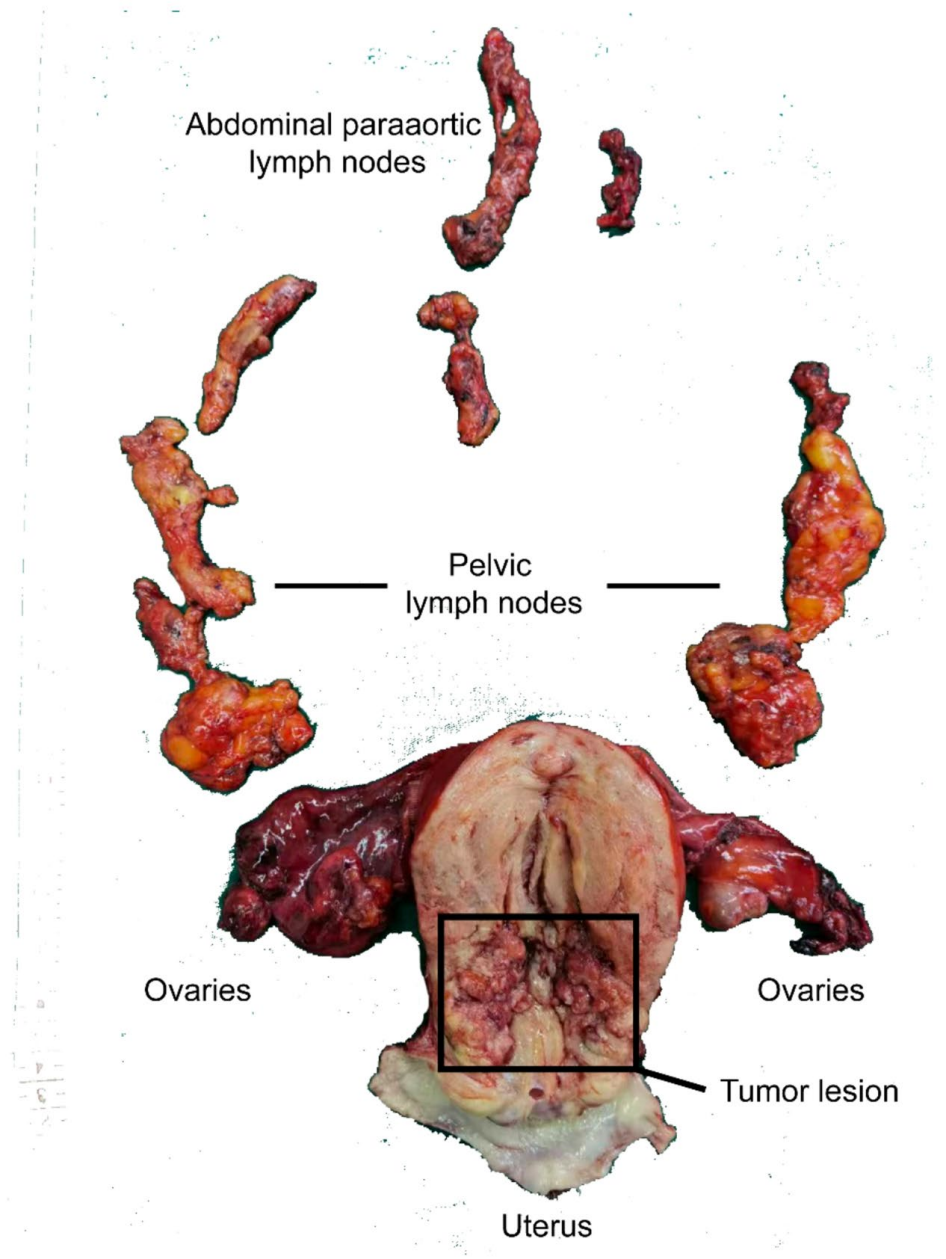
Gross photograph of the surgical specimen. The radical hysterectomy specimen (uterus with bilateral adnexa) and dissected pelvic lymph nodes are shown

**Fig. 3:**
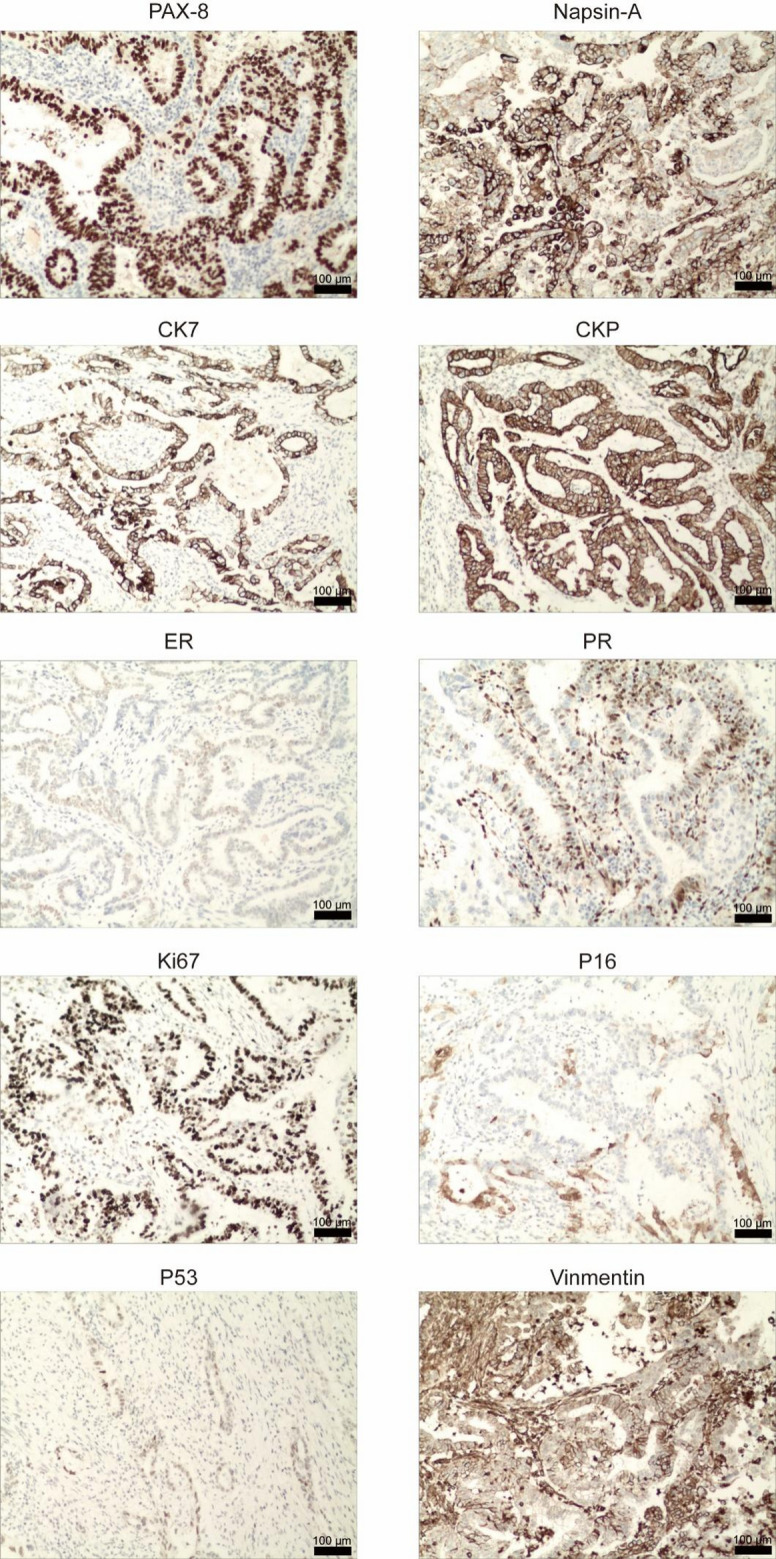
Immunohistochemical reagents and images

The patient subsequently underwent a radical hysterectomy with bilateral salpingo-oophorectomy and systematic pelvic and para-aortic lymphadenectomy. The surgical specimen contained a cauliflower-like tumor measuring approximately 4.0 × 4.5 × 1.5 cm, involving both the endocervical canal and cervix (Fig. 2). Final histopathology confirmed a moderately to poorly differentiated adenocarcinoma with clear cell features, demonstrating deep stromal invasion (> 1/2 of the cervical wall thickness) (Fig. 3). All resection margins (parametria and vaginal cuff) and resected lymph nodes were free of malignancy.

The postoperative IHC profile is summarized in Table 1. The tumor cells were positive for pan-CK, PAX-8, and CK. There was focal positivity for Vimentin, Napsin-A, ER, and PR. p16 showed patchy positivity, while p53 was partially positive (wild-type pattern). The Ki-67 proliferation index was 70% in hotspot areas. Stains for CEA, CD10, GATA3, MUC6, and TTF-1 were negative.

**Table 1:** Immunohistochemical reagents and results

Marker	Result	Pattern
pan-CK	Positive	Diffuse cytoplasmic
CEA	Negative	Not applicable
Vimentin	Positive	Focal cytoplasmic
p16	Positive	Patchy (non-block type)
TP53	Positive	Wild-type scattered
PAX-8	Positive	Diffuse nuclear
ER	Positive	Focal weak nuclear
PR	Positive	Focal weak nuclear
Napsin-A	Positive	Focal cytoplasmic
CD10	Negative	Not applicable
Ki67	Positive (70%)	High in hotspot areas
GATA3	Negative	Not applicable
MUC6	Negative	Not applicable
CK7	Positive	Cytoplasmic
TTF-1	Negative	Not applicable

CKp pan-cytokeratin, CEA carcinoembryonic antigen, ER estrogen receptor, PR progesterone receptor, PAX Paired Box Gene, CK cytokeratin, MUC Mucin, TTF thyroid tanscription factor

Given the presence of high-risk features (deep stromal invasion), the patient received three cycles of adjuvant chemotherapy with paclitaxel plus cisplatin. Serial monitoring of tumor markers postoperatively showed a significant decline to within normal limits: CA-125 (18.0 U/mL and 18.7 U/mL) and CA19-9 (25.5 U/mL and 4.1 U/mL). Following chemotherapy, the patient was transferred to the radiation oncology department to continue subsequent adjuvant radiotherapy.

## Discussion

The diagnosis and management of this case underscore several important clinicopathological characteristics of sporadic CCAC. Clear cell adenocarcinoma of the cervix (CCAC) represents a rare histological subtype, constituting approximately 4% to 9% of all cervical adenocarcinomas [5–7]. The epidemiological landscape of CCAC has been profoundly shaped by the historical association with in utero exposure to diethylstilbestrol (DES). Prenatal DES exposure is a well-established risk factor that substantially increases the relative risk of developing CCAC in the vagina and cervix [3, 8]. Approximately 80% of DES-exposed patients are diagnosed between the ages of 15 and 31 years, with the external cervix and upper anterior vaginal wall most frequently involved, their 5-year survival rate is about 86.1%. In contrast, the patients without DES exposure, the onset age is mostly 65–80 years old, primarily involving the endocervix, and its 5-year survival rate is about 81.2% [8, 9].Notably, our case involves a 46-year-old perimenopausal female with no history of DES exposure, whose onset age falls right between these two typical peaks, presenting an atypical epidemiological manifestation. Furthermore, the patient tested negative for high-risk human papillomavirus (HPV), which is consistent with the biological characteristic of CCAC being an HPV-independent tumor, and also serves as an important feature distinguishing it from HPV-driven common cervical endocervical adenocarcinoma [10].

The diagnosis in this clinically atypical case was ultimately confirmed by its distinctive immunohistochemical profile. The tumor exhibited diffuse positivity for broad-spectrum cytokeratin (pan-CK) along with nuclear PAX-8 and focal cytoplasmic napsin-A, a profile strongly supportive of a Müllerian clear cell carcinoma. Critically, the p16 expression was only patchy and non-block, which contrasts sharply with the diffuse, strong positivity characteristic of HPV-associated endocervical adenocarcinomas and confirms its HPV-independent status. The wild-type (scattered) p53 staining pattern helped argue against other high-grade mimics, such as serous carcinoma. While focal, weak positivity for ER and PR was noted—a finding documented in a subset of CCACs and reflective of phenotypic heterogeneity—it does not challenge the diagnosis. The high Ki-67 index (70%) correlated with the tumor’s aggressive potential. Furthermore, negative results for CEA, MUC6, GATA3, and TTF-1 effectively excluded key differential diagnoses, including gastric-type [11] adenocarcinoma and metastases from the breast [12] or lung [13].

With the cessation of DES use, the majority of contemporary CCACs are considered sporadic, necessitating exploration of alternative etiologies. Prevailing hypotheses often focus on potential precursor lesions. Notably, some studies have proposed that CCAC may arise from Müllerian-derived tissues with metaplastic potential, such as cervical endometriosis or tubo-endometrioid metaplasia (TEM) [1], providing a plausible histogenetic pathway for sporadic tumors. However, in our patient, no such precursor lesion was identified upon thorough histological examination. This absence, coupled with the patient’s perimenopausal age at presentation, leads us to cautiously consider other contributing factors. Hormonal fluctuations characteristic of the perimenopausal transition could theoretically influence the local microenvironment, potentially playing a role in tumor development or progression. Furthermore, the possibility of a bidirectional relationship—whereby a nascent tumor might itself alter the local hormonal milieu—cannot be excluded, making the exact nature of this association complex. It must be emphasized that these considerations remain speculative, and a causative link cannot be established from a single case.

Beyond morphological precursors, investigations into the genetic underpinnings of CCAC have begun to reveal alterations in specific molecular pathways. Notably, studies have reported a deficiency in DNA mismatch repair (MMR) protein expression in a small but significant subset (approximately 5%) of CCAC cases [14, 15]. In the clinical evaluation of our patient, the absence of personal or family history suggestive of a hereditary cancer syndrome did not warrant further testing for MMR deficiency. This clinical judgment is supported by the tumor’s immunohistochemical profile, specifically the wild-type pattern of p53 expression, which is atypical for MMR-deficient cancers. Therefore, the combined clinical and pathological findings are most consistent with a sporadic, rather than a hereditary, etiology, aligning with the majority of CCACs which arise from complex, multifactorial pathways.

In summary, we report a case of cervical clear cell adenocarcinoma in a 46-year-old perimenopausal woman that is remarkable for its atypical clinical presentation, straddling the conventional age peaks and lacking all major known risk factors. The definitive diagnosis was secured through a characteristic immunohistochemical profile featuring PAX-8/napsin-A co-expression and a non-diffuse p16 pattern. This case underscores that sporadic CCAC can emerge in a clinical vacuum without clear precursors, potentially influenced by physiologic states like perimenopausal hormonal transition. It reinforces the critical role of meticulous histopathologic examination supplemented by a tailored immunohistochemical panel in arriving at an accurate diagnosis, especially for rare tumors that can mimic more common entities. Reporting such atypical instances is vital to deepening the collective understanding of the clinical and biological spectrum of this rare malignancy.

## Data Availability

The datasets analyzed in this study are publicly available from the National Health and Nutrition Examination Survey (NHANES) repository, hosted by the Centers for Disease Control and Prevention (CDC), and can be accessed at: https://www.cdc.gov/nchs/nhanes.
